# Comparison of Management and Outcomes in *ERBB2*-Low vs *ERBB2*-Zero Metastatic Breast Cancer in France

**DOI:** 10.1001/jamanetworkopen.2022.31170

**Published:** 2022-09-15

**Authors:** Ombline de Calbiac, Amélie Lusque, Audrey Mailliez, Thomas Bachelot, Lionel Uwer, Marie-Ange Mouret-Reynier, George Emile, Christelle Jouannaud, Anthony Gonçalves, Anne Patsouris, Véronique Diéras, Marianne Leheurteur, Thierry Petit, Paul Cottu, Jean-Marc Ferrero, Véronique D'Hondt, Isabelle Desmoulins, Joana Mourato-Ribeiro, Anne-Laure Martin, Jean-Sébastien Frenel

**Affiliations:** 1 Department of Medical Oncology, Institut de Cancérologie de l’Ouest Nantes and Angers, Saint-Herblain, France; 2 Department of Biostatistics, Institut Claudius Regaud–IUCT Oncopole, Toulouse, France; 3 Department of Medical Oncology, Centre Oscar Lambret, Lille, France; 4 Department of Medical Oncology, Centre Léon Bérard, Lyon, France; 5 Department of Medical Oncology, Institut de Cancérologie de Lorraine, Vandoeuvre-lès-Nancy, France; 6 Department of Medical Oncology, Centre Jean Perrin, Clermont Ferrand, France; 7 Department of Medical Oncology, Centre François Baclesse, Caen, France; 8 Department of Medical Oncology, Institut de Cancérologie Jean-Godinot, Reims, France; 9 Department of Medical Oncology, Institut Paoli-Calmettes, Marseille, France; 10 Department of Medical Oncology, Institut de Cancérologie de l’Ouest Nantes and Angers, Angers, France; 11 Department of Medical Oncology, Centre Eugène Marquis, Rennes, France; 12 Department of Medical Oncology, Centre Henri Becquerel, Rouen, France; 13 Department of Medical Oncology, Centre Paul Strauss, Strasbourg, France; 14 Department of Medical Oncology, Institut Curie, Paris and Saint-Cloud, France; 15 Department of Medical Oncology, Centre Antoine Lacassagne, Nice, France; 16 Department of Medical Oncology, Institut du Cancer de Montpellier, Montpellier, France; 17 Department of Medical Oncology, Centre Georges-François Leclerc, Dijon, France; 18 Department of Cancer Medicine, Gustave Roussy, Villejuif, France; 19 Health Data and Partnerships Department, Unicancer, Paris, France

## Abstract

**Importance:**

*ERBB2*-low (ie, *ERBB2* immunohistochemistry score of 1+ or 2+ in the absence of *ERBB2* gene amplification) breast cancer (BC) is a new entity, with emerging dedicated treatments. Little is known about its prognosis and response to conventional therapy compared with *ERBB2*-zero breast tumors (ie, those with an immunohistochemistry score of 0).

**Objective:**

To compare the outcomes for patients with *ERBB2*-low metastatic BC (MBC) with those of patients with *ERBB2*-zero MBC.

**Design, Setting, and Participants:**

This cohort study was conducted from the Epidemiological Strategy and Medical Economics MBC platform and included patients with MBC treated between 2008 and 2016 in 18 French comprehensive cancer centers. The data analysis was conducted from July 16, 2020, to April 1, 2022.

**Main Outcomes and Measures:**

The main outcome was overall survival (OS), and the secondary outcome was progression-free survival under first-line treatments (PFS1).

**Results:**

The median (range) age was 60.0 (22.0-103.0) years. Among 15 054 patients with MBC, 4671 (31%) had *ERBB2*-low MBC and 10 383 (69%) had *ERBB2*-zero MBC. The proportion of *ERBB2*-low cancers was higher among patients with hormone receptor–positive MBC than those with hormone receptor–negative disease (4083 patients [33.0%] vs 588 patients [21.0%]). With a median follow-up of 49.5 months (95% CI, 48.6-50.4 months), the median OS of the *ERBB2*-low group was 38.0 months (95% CI, 36.4-40.5 months) compared with 33.9 months (95% CI, 32.9-34.9 months) for the *ERBB2*-zero group (*P* < .001). After adjustment for age, visceral metastases, number of metastatic sites, de novo disease, period of care, and hormone receptor status, patients with *ERBB2*-low MBC had slightly better OS compared with patients with *ERBB2*-zero MBC (adjusted hazard ratio, 0.95; 95% CI, 0.91-0.99; *P* = .02). In contrast, PFS1 did not differ by *ERBB2* status (adjusted hazard ratio, 0.99; 95% CI, 0.95-1.02; *P* = .45). No significant differences in OS and PFS1 were observed in multivariate analyses by hormone receptor status and types of frontline treatment.

**Conclusions and Relevance:**

In this large cohort study, patients with *ERBB2*-low MBC had a slightly better OS than those with completely *ERBB2*-zero tumors, but identical PFS1, which could help guide treatment selection.

## Key Points

**Question:**

Does *ERBB2*-low metastatic breast cancer have a different prognosis than *ERBB2*-zero metastatic breast cancer?

**Findings:**

In this cohort study that enrolled 15 054 patients with metastatic breast cancer from the Epidemiological Strategy and Medical Economics database, patients with *ERBB2*-low metastatic breast cancer had longer survival than did patients with *ERBB2*-zero metastatic breast cancer (38.0 vs 33.9 months).

**Meaning:**

These findings suggest that *ERBB2*-low metastatic breast cancer has a slightly better prognosis compared with *ERBB2*-zero cancer, which could help guide treatment selection.

## Teaser

This cohort study examines outcomes for patients with *ERBB2*-low metastatic breast cancer compared with those of patients with *ERBB2*-zero cancer.

## Introduction

The *ERBB2*-low breast cancer (BC) subtype is a newly proposed subtype for patients with tumors that have an immunohistochemistry (IHC) assay score of 1+ or 2+ without *ERBB2* gene amplification.^1,2^ In clinical practice, in contrast to *ERBB2*-positive BC (ie, IHC score 3+ or IHC score 2+ with *ERBB2* gene amplification), patients with these tumors are currently not candidates for anti-*ERBB2* targeted therapy.^3,4^ However, interest in this subgroup is growing with the emergence of antibody-conjugated drugs that have shown promising antitumor activity for this subgroup.^5,6,7^ Even if patients with *ERBB2*-low tumors are treated in the same manner as those with *ERBB2*-zero tumors (ie, tumors with IHC score 0) in accordance with the current guidelines,^3,8^ some evidence has suggested that these tumors are distinct. Some retrospective studies in early BC have suggested a worse prognosis,^9,10,11^ improved clinical outcomes,^12^ or similar clinical outcomes^13,14^ compared with *ERBB2*-zero disease. Regarding metastatic disease, very few and contradictory data are available.^15,16^ Considering the new emerging therapies for *ERBB2*-low BC, especially in a metastatic setting, a better description of the epidemiology, response to treatment, and outcomes of that population seems important. Our goal was to provide a comprehensive analysis of *ERBB2*-low metastatic BC (MBC) management and prognosis compared with *ERBB2*-zero MBC in, to our knowledge, the largest cohort to date.

## Methods

### Study Design

This noninterventional, retrospective cohort study aimed to describe the management and outcomes of patients with *ERBB2*-zero MBC selected from the Epidemiological Strategy and Medical Economics (ESME) MBC database. The ESME MBC database is a multicenter database that uses a retrospective data collection process (18 French comprehensive cancer centers over 20 sites). This database compiles data from patients’ electronic medical records. Patients who started a first-line anticancer treatment for MBC in any of the 18 cancer centers that participated in the ESME Research Program from January 1, 2008, to December 31, 2016, were enrolled. In the present study, we specifically selected patients whose tumor was *ERBB2*-zero or *ERBB2*-low, as defined later in this article. The data were compiled until the cutoff date (January 24, 2020), death, or date of last contact (if lost to follow-up). The analysis was approved by an independent ethics committee (Comité De Protection Des Personnes Sud-Est). No formal dedicated informed consent was required; however, all patients had approved the reuse of their electronically recorded data. In compliance with French regulations, the ESME-MBC database was authorized by the French data protection authority. Moreover, in compliance with the applicable European regulations, a complementary authorization was obtained on October 14, 2019, regarding the ESME Research Data Warehouse. This study follows the Strengthening the Reporting of Observational Studies in Epidemiology (STROBE) reporting guideline.

### Tumor Subtype Assessment

Standard guidelines were applied to any analysis performed within the ESME database. *ERBB2* and hormone receptor statuses were derived from existing results on metastatic tissue sampling if available or, if unavailable, from the last sampling of early disease. If 2 or more histologic reports were available on the same date, a positive status was considered dominant. No central review was executed. BC was hormone receptor positive if estrogen receptor or progesterone receptor expression was 10% or higher according to IHC, as per European guidelines.^17^

*ERBB2* testing relied on the combination of IHC score and fluorescence in situ hybridization (FISH) or chromogenic in situ hybridization (CISH) if necessary. On the basis of the completeness, intensity, and percentage of cells in which the staining is identified, *ERBB2* IHC is scored from 0 to 3+. In the case of an equivocal result (score 2+), FISH or CISH is performed. An *ERBB2*-zero score corresponded to an IHC score of 0, whereas an *ERBB2*-low score corresponded to an IHC score of 1+ or 2+ with negative FISH or CISH findings.

### Objectives and End Points

The primary objective of the present study was to compare the overall survival (OS) of patients with *ERBB2*-low MBC with that of patients with *ERBB2*-zero MBC in the overall population and in the hormone receptor–positive and hormone receptor–negative population subtypes. The secondary objectives were to compare progression-free survival under first-line treatments (PFS1) between these groups and to describe the patterns of treatment and evolution of the *ERBB2* status between early disease and metastatic disease, if available. OS was the primary end point, defined as the delay between metastatic diagnosis and death from any cause. PFS1 was defined as the time between the starting date of first-line treatment and the date of first disease progression or date of death. The main method for handling missing time-to-event data was censoring. Patients who were still alive and without progression at the time of the analysis were censored at their last follow-up. A treatment line was defined as a given therapeutic strategy that was set up until disease progression or death; therefore, it may have involved multiple treatments, including chemotherapy, targeted agents, or endocrine therapy. De novo metastatic disease was defined as the presence of metastasis at the time or within 6 months (180 days) from the primary tumor diagnosis. Disease progression was defined as the appearance of a new metastatic site or progression of pre-existing metastases at least 1 month after the start of treatment.

### Statistical Analysis

The data analysis was conducted from July 16, 2020, to April 1, 2022. Demographic characteristics, clinicopathological characteristics, and first-line treatment modalities of *ERBB2*-low MBC are presented for the overall population and by *ERBB2* and hormone receptor status using commonly used statistics. Continuous variables were summarized using the median, minimum, maximum, and number of missing data. Qualitative variables have been summarized for the overall population and by IHC subgroups using counts, percentages, and the number of missing data. Differences between groups were assessed using a χ^2^ or Fisher exact test for qualitative variables and Kruskal-Wallis test for continuous variables. The Kaplan-Meier method was used to estimate survival rates and median survival times in the overall population and by groups. Comparisons of *ERBB2*-low and *ERBB2*-zero MBC were performed using a 2-sided log-rank test. Univariable analysis was performed to identify the risk factors associated with OS and PFS1. Multivariable analysis was performed using a Cox proportional hazards model to evaluate the association between *ERBB2* expression and OS or PFS1 adjusting for risk factors. Subgroups analyses were performed by hormone receptor status and treatment types. All statistical tests were 2-sided, and *P* < .05 was considered significant. Statistical analyses were performed using Stata statistical software version 16 (StataCorp).

## Results

### Patient and Tumor Characteristics Among Patients With *ERBB2*-Low MBC vs *ERBB2*-Zero MBC

**Figure 1.**
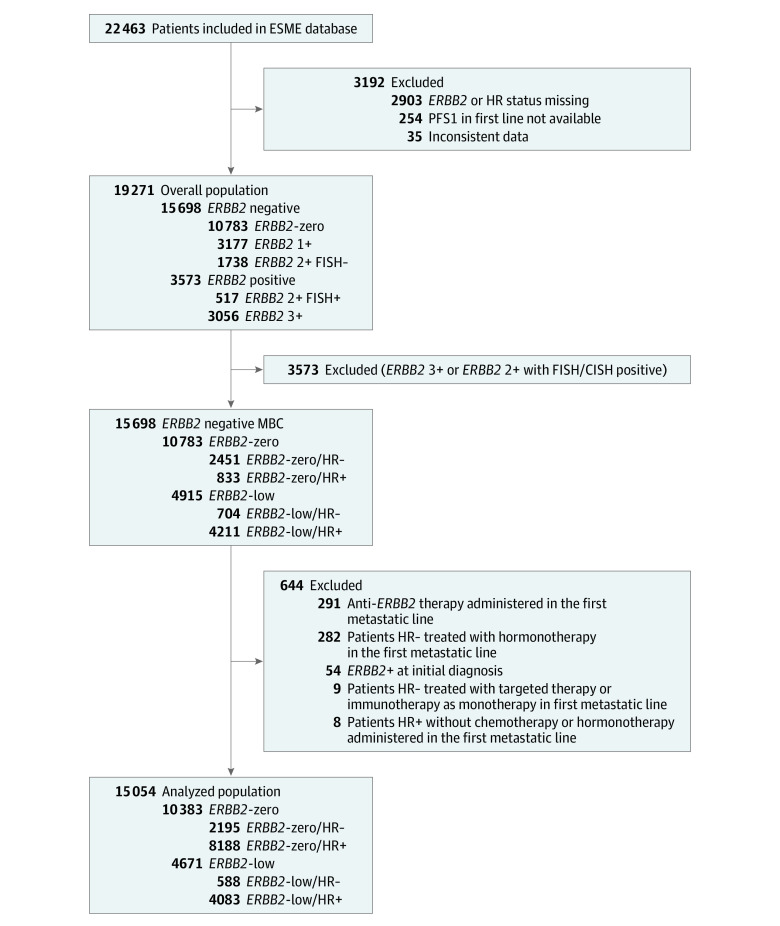
Participant Enrollment Flowchart CISH indicates chromogenic in situ hybridization; ESME, Epidemiological Strategy and Medical Economics; FISH, fluorescence in situ hybridization; HR, hormone receptor; MBC, metastatic breast cancer; PFS1, progression-free survival under first-line treatments.

**Figure 2.**
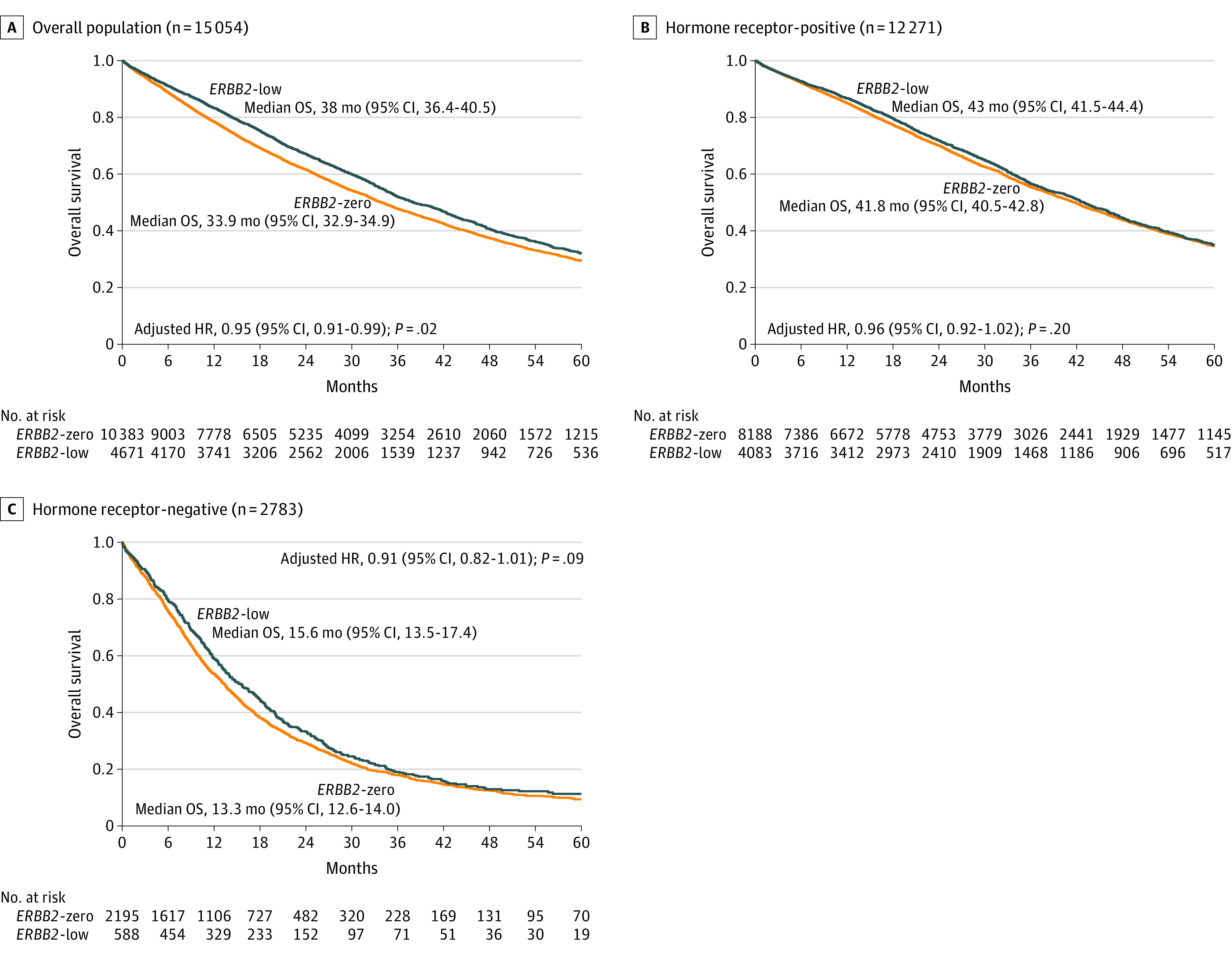
Kaplan-Meier Analysis for Overall Survival (OS) According to *ERBB2* Status (*ERBB2*-Low vs *ERBB2*-Zero) Disease outcomes were compared among the populations of patients with *ERBB2*-low and *ERBB2*-zero tumors, with separate analyses for the overall population (A), hormone receptor–positive tumors (B), and hormone receptor–negative tumors (C). HR indicates hazard ratio.

A total of 15 054 patients in the database matched the inclusion criteria. The median (range) age was 60.0 (22.0-103.0) years. A study flowchart is shown in Figure 1. The *ERBB2* status was identified as *ERBB2*-low in 4671 patients (31%) and as *ERBB2*-zero in 10 383 patients (69%). In the hormone receptor–positive population (12 271 patients), 4083 patients (33.0%) had *ERBB2*-low tumors, whereas this number was 588 (21.0%) in the triple-negative BC (TNBC) population (2783 patients). Patients’ characteristics according to *ERBB2* expression are shown in the Table. In the *ERBB2*-low group, tumor grades were mainly II and III (89.6%), similar to the *ERBB2*-zero group. Patients with *ERBB2*-low MBC had more frequent de novo metastatic disease compared with patients in the *ERBB2*-zero group (1742 patients [37.3%] vs 2889 patients [27.8%]) (Figure 2).

**Table.** Patient Characteristics and Treatments According to *ERBB2* Status in the Overall Population and by Hormone Receptor Subgroup

Characteristics	Patients, No. (%) (N = 15 054)					
	*ERBB2*-zero			*ERBB2*-low		
	Total (n = 10 383)	Hormone receptor positive (n = 8188)	Hormone receptor negative (n = 2195)	Total (n = 4671)	Hormone receptor positive (n = 4083)	Hormone receptor negative (n = 588)
Age at MBC diagnosis, median (range), y	60.0 (22.0-96.0)	61.0 (22.0-96.0)	54.0 (22.0-93.0)	61.0 (22.0-103.0)	62.0 (23.0-103.0)	59.0 (22.0-94.0)
Age range at MBC diagnosis, y						
<50	2580 (24.8)	1751 (21.4)	829 (37.8)	1016 (21.8)	872 (21.4)	144 (24.5)
50-70	5332 (51.4)	4307 (52.6)	1025 (46.7)	2433 (52.1)	2114 (51.8)	319 (54.3)
>70	2471 (23.8)	2130 (26.0)	341 (15.5)	1222 (26.2)	1097 (26.9)	125 (21.3)
Sex						
Male	87 (0.8)	83 (1.0)	4 (0.2)	54 (1.2)	53 (1.3)	1 (0.2)
Female	10296 (99.2)	8105 (99.0)	2191 (99.8)	4617 (98.8)	4030 (98.7)	587 (99.8)
Menopausal status						
No	3148 (30.6)	2178 (26.9)	970 (44.3)	1247 (27.0)	1057 (26.2)	190 (32.4)
Yes	7148 (69.4)	5927 (73.1)	1221 (55.7)	3370 (73.0)	2973 (73.8)	397 (67.6)
Missing^a^	87	83	4	54	53	1
Primary tumor grade						
I	1052 (12.0)	1020 (14.8)	32 (1.7)	425 (10.4)	412 (11.6)	13 (2.4)
II	4736 (53.9)	4182 (60.7)	554 (29.2)	2385 (58.4)	2211 (62.2)	174 (32.8)
III	3003 (34.2)	1689 (24.5)	1314 (69.2)	1275 (31.2)	931 (26.2)	344 (64.8)
Missing^a^	1592	1297	295	586	529	57
Histological subtype						
Invasive ductal	7665 (74.4)	5803 (71.5)	1862 (85.3)	3580 (77.4)	3090 (76.4)	490 (84.0)
Invasive lobular	1553 (15.1)	1473 (18.1)	80 (3.7)	613 (13.2)	575 (14.2)	38 (6.5)
Mixed	125 (1.2)	120 (1.5)	5 (0.2)	47 (1.0)	43 (1.1)	4 (0.7)
Other	956 (9.3)	721 (8.9)	235 (10.8)	387 (8.4)	336 (8.3)	51 (8.7)
Missing^a^	84	71	13	44	39	5
Interval between primary tumor and metastatic relapse, mo						
<6 (de novo MBC)	2889 (27.8)	2316 (28.3)	573 (26.1)	1742 (37.3)	1545 (37.8)	197 (33.5)
6-24	1498 (14.4)	676 (8.3)	822 (37.5)	527 (11.3)	339 (8.3)	188 (32.0)
>24	5987 (57.7)	5188 (63.4)	799 (36.4)	2401 (51.4)	2198 (53.8)	203 (34.5)
Missing^a^	9	8	1	1	1	0
No. of metastatic sites ≥3	2177 (21.0)	1615 (19.7)	562 (25.6)	1086 (23.2)	944 (23.1)	142 (24.1)
Type of metastases						
Visceral metastases	5798 (55.8)	4295 (52.5)	1503 (68.5)	2612 (55.9)	2240 (54.9)	372 (63.3)
Central nervous system	628 (6.0)	331 (4.0)	297 (13.5)	229 (4.9)	169 (4.1)	60 (10.2)
Bone	6172 (59.4)	5413 (66.1)	759 (34.6)	2962 (63.4)	2748 (67.3)	214 (36.4)
Lung	2479 (23.9)	1673 (20.4)	806 (36.7)	1170 (25.0)	970 (23.8)	200 (34.0)
Metastatic nodes	2945 (28.4)	2012 (24.6)	933 (42.5)	1414 (30.3)	1161 (28.4)	253 (43.0)
Liver	2697 (26.0)	2080 (25.4)	617 (28.1)	1278 (27.4)	1123 (27.5)	155 (26.4)
Treatment for primary tumor (in patients with metastatic relapse, n = 10 413)						
Chemotherapy or targeted therapy	5519 (73.7)	4052 (69.1)	1467 (90.5)	2085 (71.2)	1739 (68.5)	346 (88.5)
Adjuvant endocrine therapy	5107 (68.2)	5018 (85.6)	89 (5.5)	2165 (73.9)	2128 (83.9)	37 (9.5)
Radiotherapy	6651 (88.9)	5210 (88.8)	1441 (88.9)	2620 (89.5)	2271 (89.5)	349 (89.3)
First-line treatment of metastatic disease						
Endocrine therapy	3859 (37.2)	3859 (47.1)	NA	1812 (38.8)	1812 (44.4)	NA
Chemotherapy with endocrine therapy	2759 (26.6)	2759 (33.7)	NA	1514 (32.4)	1514 (37.1)	NA
Chemotherapy without endocrine therapy	3765 (36.2)	1570 (19.2)	2195 (100.0)	1345 (28.8)	757 (18.5)	588 (100.0)

Abbreviations: MBC, metastatic breast cancer; NA, not applicable.

^a^ Missing data were not included in the calculations of percentages.

### Outcomes of Patients With *ERBB2*-Low vs *ERBB2*-Zero MBC

The median follow-up for the entire population was 49.5 months (95% CI, 48.6-50.4 months) and was similar for *ERBB2*-zero and *ERBB2*-low subgroups. Patients with *ERBB2*-low MBC had significantly better OS compared with the *ERBB2*-zero group (median OS, 38.0 months [95% CI, 36.4-40.5 months] vs 33.9 months [95% CI, 32.9-34.9 months]; *P* < .001). The multivariable analysis adjusted for age, visceral metastases, number of metastases, de novo metastatic disease, period of care, and hormone receptor status confirmed that patients with *ERBB2*-low MBC had an independently better OS compared with patients with *ERBB2*-zero MBC (adjusted hazard ratio [HR], 0.95; 95% CI, 0.91-0.99; *P* = .02).

### Management and Outcomes of the Hormone Receptor–Positive Population According to *ERBB2* Status

A total of 4083 patients (33.0%) had *ERBB2*-low, hormone receptor–positive disease, and 8188 (67.0%) patients had *ERBB2*-zero, hormone receptor–positive disease. The median (range) age was similar in both groups (61.0 [22.0-103.0] years for the *ERBB2*-low group vs 61.0 [22.0-96.0] years for the *ERBB2*-zero group) as well as menopausal status, tumor grade, and number of metastatic sites (Table). De novo metastatic disease was again more frequent in the *ERBB2*-low group compared with the *ERBB2*-zero group (1545 patients [37.8%] vs 2316 patients [28.3%]). The rate of visceral metastases was 53.3% (6535 patients) without any significant difference between the groups. In patients with metastatic relapse (10 413 patients), 5791 patients (68.9%) had received previous adjuvant chemotherapy, 7481 patients (89.0%) had received radiotherapy, and 7146 patients (85.1%) had received endocrine therapy, without significant differences between both groups. The median OS was similar for both groups: 43.0 months (95% CI, 41.5-44.4 months) for the *ERBB2*-low group and 41.8 months (95% CI, 40.5-42.8 months) for the *ERBB2*-zero group (adjusted HR, 0.96; 95% CI, 0.92-1.02; *P* = .17) (Table and Figure 2B).

**Figure 3.**
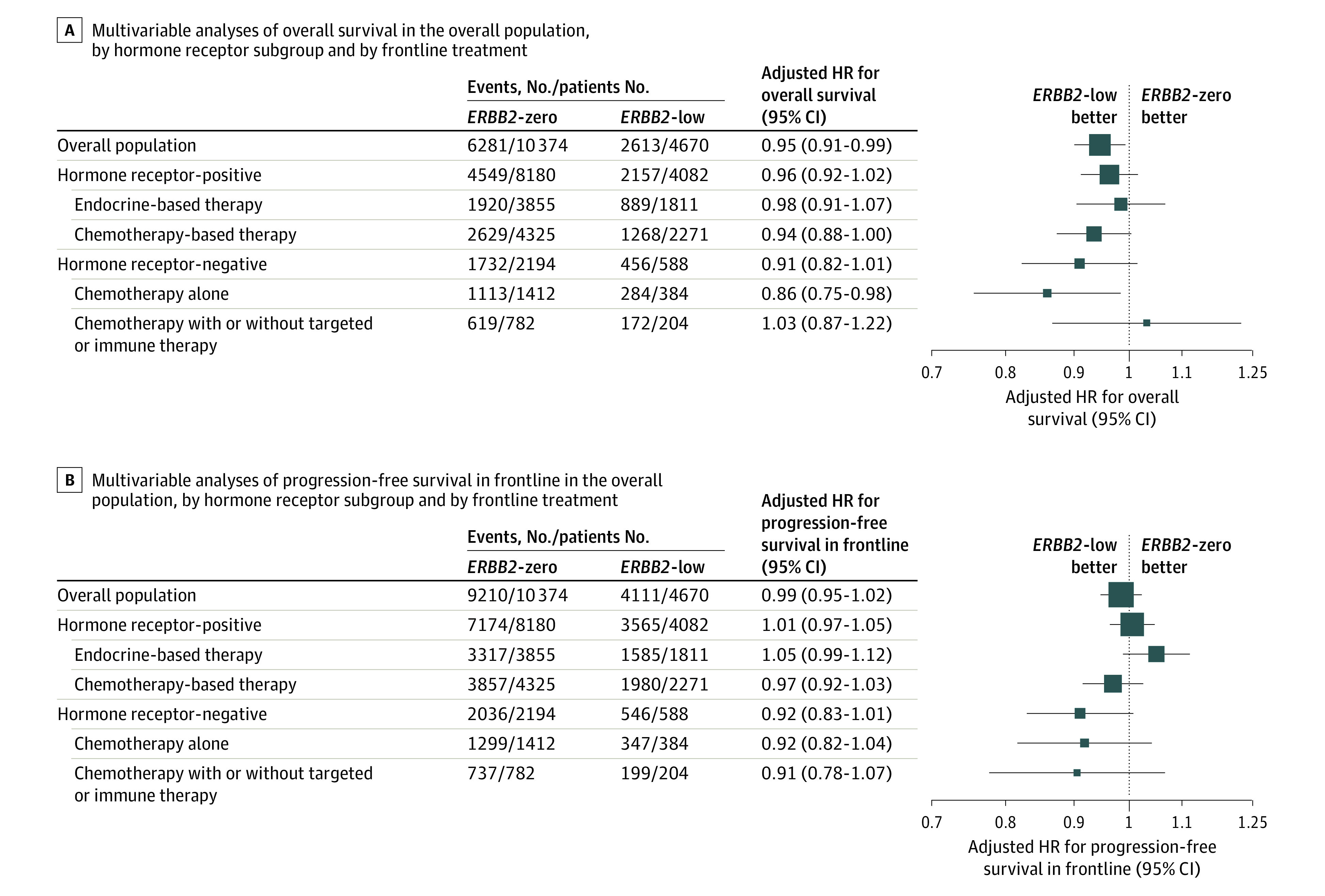
Multivariable Analyses of Overall Survival and Progression-Free Survival in Frontline in the Overall Population, by Hormone Receptor Subgroup and by Frontline Treatment The forest plots show the adjusted hazard ratio (HR) of *ERBB2*-low metastatic breast cancer compared with *ERBB2*-zero cancer.

The patterns of frontline treatment for metastatic disease were similar between the groups, with 1812 patients (44.4%) in the *ERBB2*-low group and 3859 patients (47.1%) in the *ERBB2*-zero group receiving endocrine-based therapy, and 2271 patients (55.6%) in the *ERBB2*-low group and 4329 patients (52.9%) in the *ERBB2*-zero group receiving chemotherapy. These patients were treated between 2008 and 2016; thus, only a few received frontline CDK4-6 inhibitors (63 patients). The univariate analysis indicated that patients with *ERBB2*-low and *ERBB2*-zero MBC had a similar PFS1 under endocrine therapy (median PFS1, 10.8 months [95% CI, 10.1-11.5 months] vs 10.6 months [95% CI, 10.1-11.3 months]). Similarly, no significant difference was found for frontline chemotherapy between the groups (median PFS1, 11.0 months [95% CI, 10.4-11.5 months] vs 10.0 months [95% CI, 9.6-10.5 months]). Similar results were found in multivariable analyses. PFS1 did not differ by *ERBB2* status (adjusted HR, 0.99; 95% CI, 0.95-1.02; *P* = .45) (Figure 3). In univariate analyses, the median OS rate was similar between the groups for patients treated with frontline endocrine therapy. However, patients with *ERBB2*-low MBC who received frontline chemotherapy had slightly longer OS than patients with *ERBB2*-zero MBC (median OS, 39.7 months [95% CI, 37.3-42.0 months] vs 36.8 months [95% CI, 35.1-38.3 months]), but the difference was not significant (adjusted HR, 0.94; 95% CI, 0.88-1.00; *P* = .06) (eTable 1 in the Supplement).

### Management and Outcome Within the TNBC Population According to *ERBB2* Status

A total of 588 patients (21%) had *ERBB2*-low, hormone receptor–negative disease, and 2195 patients (79%) had *ERBB2*-zero, hormone receptor–negative disease. Patients with *ERBB2*-low MBC were slightly older than those with *ERBB2*-zero MBC (median [range] age, 59.0 [22.0-94.0] years vs 54.0 [22.0-93.0] years) (Table and Figure 2C). First-line treatment included chemotherapy alone or in combination with targeted therapy–immune therapy in 380 patients (64.6%) with *ERBB2*-low MBC and 777 patients (35.4%) with *ERBB2*-zero MBC. Of note, bevacizumab represented 91.9% (901 therapies) of all targeted therapies. In the univariate analysis, patients with *ERBB2*-low MBC had better PFS1 with frontline chemotherapy-based therapy compared with the *ERBB2*-zero group (median PFS1, 5.3 months [95% CI, 4.8-5.7 months] vs 4.6 months [95% CI, 4.4-4.9 months]; *P* = .009) (eTable 2 in the Supplement). Multivariable analysis including age, visceral metastases, number of metastases, de novo metastatic disease, period of care, and hormone receptor status did not confirm this observation (adjusted HR, 0.92; 95% CI, 0.83-1.01; *P* = .07) (Figure 3). Patients with *ERBB2*-low MBC also had longer OS compared with patients with *ERBB2*-zero MBC (median, 15.6 months [95% CI, 13.5-17.4 months] vs 13.3 months [95% CI, 12.6-14.0 months]; *P* = .04) (eTable 2 in the Supplement). However, in the multivariable Cox model analysis, including age, type and number of metastases, de novo metastatic diseases, and period of care, *ERBB2*-low status was not significantly associated with OS (adjusted HR, 0.91; 95% CI, 0.82-1.01; *P* = .09) (Figure 3).

### Evolution of Low *ERBB2* Expression Between Early and Advanced-Stage BC

**Figure 4.**
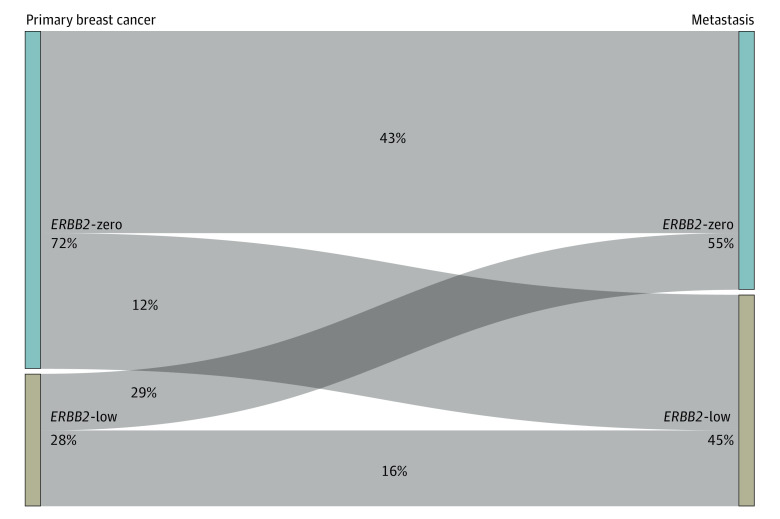
*ERBB2* Discordance From Primary Breast Cancer to Metastasis in the ESME Database The overall discordance rate from primary tumor to metastasis was 40.9% (1005 tumors).

*ERBB2* status was determined for the metastatic tissue in 1423 cases (9.5%) and for the primary tumor (when no biopsy of metastatic tissue was performed) in 13 631 cases (90.5%) (Figure 4). A discordant *ERBB2* status (low vs zero) between primary vs metastatic assessment was found in 411 of 1005 patients (40.9%) with primary and metastatic tissue available. A total of 290 patients (28.9%) with an *ERBB2*-zero primary tumor switched to *ERBB2*-low status at metastasis. Conversion from *ERBB2*-low to *ERBB2*-zero was observed in 121 patients (12%). Details of discordance rate according to hormone receptor status, and impact on OS is shown in eTable 3 in the Supplement.

## Discussion

After the update of the American Society of Clinical Oncology and College of American Pathologists guidelines for *ERBB2* assessment in BC, a new category named *ERBB2*-low was proposed.^1,3^ In this cohort study, our large, unique data set allowed a comprehensive investigation of the epidemiology and the impact of this category in the context of MBC.

Distinguishing *ERBB2*-zero from *ERBB2*-low MBC has not been clinically relevant so far and, for practical purposes, these 2 groups have until recently often been combined. All large-scale reports have been based on registry studies that included patients who were treated before the establishment of this new category. Two recently published large studies^15,16^ have provided insight on this category of BC. The first study included 1378 patients with *ERBB2*-zero MBC from the MBC registry of the Austrian Study Group of Medical Tumor Therapy and reported a 44% prevalence rate for *ERBB2*-low MBC,^16^ whereas a study that used the China National Center Database reported a similar prevalence rate of 43.1%.^15^ In early BC, a pooled analysis of individual patient’s data from 2310 patients with *ERBB2*-zero BC who were included in 4 prospective neoadjuvant clinical trials resulted in a prevalence rate of 47.5% for *ERBB2*-low BC.^12^

*ERBB2*-low expression seems unstable during evolution of the disease. We observed a rate of overall discordance in the primary tumor and metastasis of 40.9%. A switch from a *ERBB2*-zero primary tumor to a *ERBB2*-low metastatic tumor was the most frequent (28.9%). Two recent Italian reports^18,19^ have highlighted the dynamics of *ERBB2* expression. A similar discordance rate was reported (38.0%), mostly represented by *ERBB2*-zero disease switching to *ERBB2*-low disease (36.4% of the *ERBB2*-zero cohort) and *ERBB2*-low disease to *ERBB2*-zero disease (41.2% of the *ERBB2*-low cohort).^18,19^ The discrepancy in *ERBB2* expression between primary and metastatic disease may result from genetic drift during tumor progression,^20^ intratumoral heterogeneity,^21^ and selective pressure of therapies with potential upregulation of *ERBB2* expression.^22,23^ This instability is important to consider if dedicated drugs are approved in the near future.

Previous reports show inconsistent data regarding the specific characteristics of *ERBB2*-low BC and whether this is a distinct biological entity. In the hormone receptor–positive BC group, *ERBB2*-low expression was 33.0% in our study and approximately the same in previous reports.^24^ We did not notice any difference in terms of age, tumor grade, and number or type of metastases. However, the proportion of de novo metastatic diseases was higher in the *ERBB2*-low population. Considering a biological point of view, a recent report by Schettini et al^14^ identified higher luminal-related gene expression levels in *ERBB2*-low, hormone receptor–positive tumors compared with *ERBB2*-zero, hormone receptor–positive tumors. In the TNBC population, the frequency of *ERBB2*-low expression has been estimated from 21.0% of the patients in our study to 35.0% in previous reports.^14,15^ We did not notice any difference in tumor grade or the number or type of metastases, whereas de novo metastatic disease was also more frequent in the *ERBB2*-low group. Recently, Agostinetto et al^13^ presented a retrospective analysis of molecular characteristics of 410 patients with primary *ERBB2*-low BC, among whom 74 had TNBC. Using the *PAM50* intrinsic subtype classification, they found a higher rate of *ERBB2*-enriched tumors in the *ERBB2*-low TNBC group compared with the *ERBB2*-zero TNBC population (13.7% vs 1.2%).^13^ Despite the high frequency of brain metastases in patients with *ERBB2*-positive MBC,^25^ no difference in brain metastasis frequency was noticed in our series according to *ERBB2* expression.

*ERBB2*-low status was an independent factor associated with longer OS in the global population of our study. Our results also suggest that hormone receptor status was a key determinant of clinical outcomes in patients with *ERBB2*-low tumors. Similarly, Li et al^15^ reported an improved OS rate for patients with *ERBB2*-low MBC compared with those with *ERBB2*-zero MBC (48.5 months vs 43.0 months; *P* = .004). A positive impact was also significant in the hormone receptor–positive group (54.9 months vs 48.1 months; *P* = .01) but not in the hormone receptor–negative subgroup (*P* = .72).^15^ On the other hand, other studies have failed to demonstrate any impact of *ERBB2*-low status in a metastatic setting.^14,16,26^ In early BC, a study^12^ enrolling 2310 patients with *ERBB2*-zero early BCs from neoadjuvant trials showed that patients with *ERBB2*-low BC had a lower pathological complete response to neoadjuvant chemotherapy compared with patients with *ERBB2*-zero tumors (29.2% vs 39.0%; *P* < .001) suggesting lower chemosensitivity.^12^ However, patients with *ERBB2*-low tumors had better OS (adjusted HR, 0.64; 95% CI, 0.48-0.86; *P* = .003).^12^

The growing interest in the *ERBB2*-low BC subgroup is associated with the rapid development of antibody-drug conjugates.^27^ Trastuzumab-deruxtecan has shown impressive results compared with standard chemotherapy with an improvement in PFS (HR, 0.50; 95% CI, 0.40-0.63; *P* < .001) and OS (HR, 0.64; 95% CI, 0.49-0.84; *P* = .001).^28^ These results confirm the opportunity of using *ERBB2* as a therapeutic vector in *ERBB2*-low disease and highlight the relevance of this emerging subtype of BC.^28^

### Limitations and Strengths

Our study had several limitations including its retrospective nature. However, the data were collected with a clinical trial–like method. Second, the *ERBB2* status was not centrally reviewed, and a recent study^29^ shows the lack of concordance between pathologists into distinguishing tumors with *ERBB2* score 0 and vs those with a score of 1+. RNA expression appears to be more sensitive than IHC, and quantitative methods may help define the levels of *ERBB2* expression required for response to anti-*ERBB2* antibodies.^30^ Third, most cases of *ERBB2* status were defined on the basis of the primary tumor. This may explain, in part, the relatively lower proportion of patients with *ERBB2*-low BC compared with the published data. At the same time, our study had strengths; for example, the ESME-MBC program represents a very large-scale ongoing multicenter cohort, with one of the largest numbers of patients with MBC ever included in a retrospective analysis for outcome estimates. The centralized data are both exhaustive and of high quality.

## Conclusions

Our study enrolled the largest cohort of patients, to our knowledge, with *ERBB2*-low MBC to provide insight on this new subgroup. Patients with *ERBB2*-low BCs had a slightly better OS than those with completely *ERBB2*-zero tumors. Correctly identifying *ERBB2*-low BC is a key challenge given emerging dedicated treatment and potential variability between pathologists and dynamics of this status during the course of the disease.

## Notes

Supplement. **eTable 1.** Median Overall Survival (OS) and Median Progression Free Survival (PFS1) for Metastatic Disease, With Frontline Endocrine Therapy and Frontline Chemotherapy, in the Hormone Receptor-Positive (HR+) Population **eTable 2.** Median Overall Survival (OS) and Median Progression Free Survival (PFS1) for Metastatic Disease, With Frontline Chemotherapy +/- Targeted Therapy, in the Hormone Receptor Negative Population **eTable 3.** Evolution of *ERBB2* Status Between Primary Tumor to Metastasis, According to Hormone Receptor Status, and Prognosis Impact on Overall Survival
