# Supplementary material for: Comparison of Management and Outcomes in ERBB2-Low vs ERBB2-Zero Metastatic Breast Cancer in France
Source: JAMA Netw Open. 2022 Sep 15;5(9):e2231170. doi: 10.1001/jamanetworkopen.2022.31170 (PMC9478776; doi:10.1001/jamanetworkopen.2022.31170)
Supplement: Supplement. — eTable 1. Median Overall Survival (OS) and Median Progression Free Survival (PFS1) for Metastatic Disease, With Frontline Endocrine Therapy and Frontline Chemotherapy, in the Hormone Receptor-Positive (HR+) Population eTable 2. Median Overall Survival (OS) and Median Progression Free Survival (PFS1) for Metastatic Disease, With Frontline Chemotherapy +/- Targeted Therapy, in the Hormone Receptor Negative Population eTable 3. Evolution of ERBB2 Status Between Primary Tumor to Metastasis, According to Hormone Receptor Status, and Prognosis Impact on Overall Survival [file jamanetwopen-e2231170-s001.pdf]

Supplemental Online Content

de Calbiac O, Lusque A, Mailliez A, et al. Comparison of management and outcomes in *ERBB2*-low vs *ERBB2*-zero metastatic breast cancer in France. *JAMA Netw Open*. 2022;5(9):e2231170. doi:10.1001/jamanetworkopen.2022.31170

eTable 1. Median Overall Survival (OS) and Median Progression Free Survival (PFS1) for Metastatic Disease, With Frontline Endocrine Therapy and Frontline Chemotherapy, in the Hormone Receptor-Positive (HR+) Population
eTable 2. Median Overall Survival (OS) and Median Progression Free Survival (PFS1) for Metastatic Disease, With Frontline Chemotherapy +/- Targeted Therapy, in the Hormone Receptor Negative Population
eTable 3. Evolution of *ERBB2* Status Between Primary Tumor to Metastasis, According to Hormone Receptor Status, and Prognosis Impact on Overall Survival

This supplemental material has been provided by the authors to give readers additional information about their work.

eTable 1. Median Overall Survival (OS) and Median Progression Free Survival (PFS1) for Metastatic Disease, With Frontline Endocrine Therapy and Frontline Chemotherapy, in the Hormone Receptor-Positive (HR+) Population

Population	N	Median OS [95% CI]	Median PFS1 [95% CI]
**ERBB2-low/HR+**			
Endocrine therapy*	1812	47.3 [43.9-49.1]	10.8 [10.1-11.5]
Chemotherapy*	2271	39.7 [37.3-42.0]	11.0 [10.4-11.5]
**ERBB2-0/HR+**			
Endocrine therapy*	3859	46.6 [44.9-48.3]	10.6 [10.1-11.3]
Chemotherapy*	4329	36.8 [35.1-38.3]	10.0 [9.6-10.5]

HR+, hormone receptor positive; OS, overall survival; PFS1, first-line progression-free survival.
*; with or without targeted therapy / immune therapy

© 2022 de Calbiac O et al. *JAMA Network Open.*

eTable 2. Median Overall Survival (OS) and Median Progression Free Survival (PFS1) for Metastatic Disease, With Frontline Chemotherapy +/- Targeted Therapy, in the Hormone Receptor Negative Population

Population	N	Median OS [95% CI]	Median PFS1 [95% CI]
**ERBB2-low/HR-**	588	15.6 [13.5-17.4]	5.3 [4.8; 5.7]
**ERBB2-0/HR-**	2195	13.3 [12.6-14.0]	4.6 [4.4-4.9]

HR-, hormone receptor negative; OS, overall survival; PFS1, first-line progression-free survival.
*; with or without targeted therapy / immune therapy

eTable 3. Evolution of *ERBB2* Status Between Primary Tumor to Metastasis, According to Hormone Receptor Status, and Prognosis Impact on Overall Survival

Change from primary tumor to metastase	Events/N	Overall survival, months [95% CI]
**Hormone receptor-positive**		
**ERBB2-0 concordant**	171 / 306	35.8 [32.7;41.1]
**ERBB2-low concordant**	74 / 143	35.8 [32.6;48.3]
**ERBB2-low → ERBB2-0**	43 / 88	47.1 [28.4;66.0]
**ERBB2-0 → ERBB2-low**	132 / 242	42.0 [34.9;47.4]
**Hormone receptor-negative**		
**ERBB2-0 concordant**	97/126	12.9 [9.3;15.7]
**ERBB2-low concordant**	14 / 19	20.2 [7.3;38.7]
**ERBB2-low → ERBB2-0**	25 / 33	11.2 [8.7;19.1]
**ERBB2-0 → ERBB2-low**	42 / 48	20.1 [16.4;23.4]

© 2022 de Calbiac O et al. *JAMA Network Open.*
